# ESTRO consensus recommendations for high dose rate interstitial liver brachytherapy^☆^

**DOI:** 10.1016/j.ctro.2026.101343

**Published:** 2026-11-01

**Authors:** Stefanie Corradini, Maya Rottler, Mateusz Bilski, Peter Hass, Kathrin Hering, Efstratios Karagiannis, Iosif Strouthos, Nikolaos Tselis, Mathias Walke, Konrad Mohnike, Max Seidensticker, Jens Ricke, Alexandra Stewart, On behalf of GEC-ESTRO

**Affiliations:** a Department of Radiation Oncology, Friedrich-Alexander-Universität Erlangen-Nürnberg, Erlangen, Germany; b Department of Radiation Oncology, University Hospital, LMU Munich, Munich, Germany; c Radiotherapy Department, Affidea Nu-med Center of Oncological Diagnostics and Therapy, Zamość, Poland; d Clinics of Radiotherapy, Medical University of Lublin, Poland; e Department of Radiation Oncology, Helios Erfurt, Campus of the Health and Medical University (HMU), Germany; f Department of Radiation Oncology, University of Leipzig, Germany; g Department of Radiation Oncology, Graubuenden, Switzerland; h Department of Radiation Oncology, German Oncology Center, European University Cyprus, Limassol, Cyprus; i Department of Radiation Oncology, University of Frankfurt, Germany; j Department of Radiation Oncology, University of Magdeburg, Germany; k DTZ, Berlin, Germany; l Department of Radiology, University Hospital, LMU Munich, Munich, Germany; m University of Surrey, UK

**Keywords:** Liver neoplasms, brachytherapy, HDR brachytherapy, Interstitial brachytherapy, Image-guided brachytherapy, Liver metastases, Hepatocellular carcinoma, Cholangiocarcinoma, Consensus recommendations

## Abstract

High dose rate (HDR) interstitial liver brachytherapy has become one of the established local ablative treatment options for selected primary and secondary liver tumours that are unsuitable for surgical resection. With its steep dose gradient and high conformality, it enables precise dose delivery to the target while effectively sparing surrounding healthy liver tissue and adjacent organs at risk.

These consensus recommendations provide comprehensive, standardised guidance on the technique and workflow of interstitial HDR liver brachytherapy, including image-guided catheter implantation, implantation techniques, and treatment planning. The recommendations further summarise available evidence on oncological outcomes, toxicities and adverse event management, and outline principles for post-treatment follow-up.

The consensus recommendations were developed by an expert panel of the GEC-ESTRO Gastrointestinal Working Group based on published evidence and collective clinical experience. They aim to harmonise practice across centres, promote safe and effective treatment delivery, and facilitate future research and data comparison in this evolving field of liver-directed radiotherapy.

## Highlights

• Patient selection and indications for primary and secondary liver tumours.
• Image-guided catheter implantation using the direct or Seldinger technique.
• Target delineation, dose planning, dose reporting and organs of interest constraints.
• Histology-specific outcomes for HCC, cholangiocarcinoma and liver metastases.
• Side effects, complication management and post-treatment follow-up.

## 1 Introduction

Interstitial high dose rate (HDR) brachytherapy (BT) is one of the local liver-directed ablative treatment modalities for selected liver tumours that are not amenable to surgical resection. With its physical properties and dosimetric advantages, interstitial brachytherapy offers an ablative treatment of primary and secondary liver malignancies, particularly in patients with oligometastatic disease [1], [2], [3].

Liver tumours comprise a heterogeneous group of malignancies, including primary liver cancers such as hepatocellular carcinoma (HCC) and cholangiocarcinoma (CCC) and secondary metastatic tumours. Liver cancer remains a major global cancer burden, with approximately 866,000 new cases worldwide in 2022 [4].

Treatment options for liver tumours include surgical resection, thermal ablation (e.g. radiofrequency or microwave ablation), transarterial therapies (e.g. transarterial chemoembolization (TACE), transarterial radioembolization (TARE)), systemic therapies and radiotherapy. Radiotherapy approaches include external beam radiotherapy (EBRT) techniques such as stereotactic body radiation therapy (SBRT) and interstitial HDR brachytherapy. Among radiotherapy modalities, interstitial HDR brachytherapy has emerged as a promising option for selected cases, especially when lesions are unsuitable for thermal ablation or when proximity to critical structures precludes other local treatments [1], [2], [3].

Compared to SBRT, brachytherapy offers superior dose conformality due to the inherent steep dose gradient. This allows high radiation doses to be delivered directly to the tumour, while significantly sparing surrounding healthy liver tissue and adjacent organs of interest (OoI). Dosimetric analyses comparing interstitial HDR brachytherapy with SBRT have demonstrated superior dose coverage and reduced dose exposure of healthy liver tissue with brachytherapy [2], [3]. As a result, treatment volumes can be kept small, allowing ultra-hypofractionated (usually single fraction) regimens that reduce overall treatment time and reduce the number of treatment visits.

Careful patient selection is essential, ideally guided by a multidisciplinary tumour board including hepatobiliary surgeons, interventional radiologists, medical oncologists, and radiation oncologists. Interstitial HDR brachytherapy is typically delivered via an afterloading technique using Iridium-192. Brachytherapy catheters are usually placed under image guidance (typically computed tomography (CT), magnetic resonance imaging (MRI) or ultrasound) and the procedure is associated with low morbidity and a minimal risk of serious complications such as major bleeding or infection, when performed by experienced radiation oncologists or interventional radiologists [5].

The purpose of this guideline is to provide recommendations for the use of interstitial HDR brachytherapy in the treatment of liver tumours. The Gastrointestinal (GI) Groupe Européen de Curiethérapie-European Society for Radiotherapy and Oncology (GEC-ESTRO) working group proposed development of these guidelines and formed a writing group consisting of clinical professionals experienced in interstitial HDR liver brachytherapy. Standardisation of terminology and treatment allows better comparison between groups and facilitates future clinical research. These recommendations are intended to support clinical decision making and standardise treatment practice.

## 2 Patient selection

Preprocedural imaging is essential for identifying liver malignancies suitable for brachytherapy. High-sensitivity imaging such as liver MRI with gadoxetic acid or positron emission tomography/computed tomography (PET/CT) improves detection of liver lesions and enables accurate overall tumour burden assessment.

All cases should be discussed in a multidisciplinary tumour board in accordance with guideline recommendations (e.g. European Society for Medical Oncology (ESMO) [6] or European Association for the Study of the Liver (EASL) [7]). Patient suitability should be assessed using performance scores (ECOG), laboratory parameters and liver function scores, such as Child–Pugh or ALBI. The blood count must be adequate for interventional procedures (e.g. international normalized ratio (INR) ≤1.5, platelets >50 × 10^9^/l, partial thromboplastin time (PTT) <50 s) and not older than one week. Adequate coagulation status is required before intervention, and anticoagulation management should follow the Society of Interventional Radiology (SIR) guidelines [8]. Allergy history and contrast-agent precautions should follow European Society of Urogenital Radiology (ESUR) recommendations [9]. A serum bilirubin level < 2 mg/dL has been used as an eligibility criterion in published liver brachytherapy series [10], [11].

#### 2.1.1 Indications and contraindications

Liver brachytherapy may be considered for patients with primary liver malignancies or oligometastatic liver disease when surgery is not feasible. Brachytherapy has shown a favourable safety profile even for centrally located liver tumours, which are often difficult to treat using other local modalities. Biliary duct complications in these cases are rare and have not been shown to negatively impact overall survival [12]. This may represent an advantage in centrally located lesions, for which other local treatment approaches can be technically challenging [13], [14]. However, certain clinical risk factors have been identified. Relative contraindications include advanced cirrhosis (Child–Pugh B or higher), ascites, and ongoing anticoagulation due to increased bleeding risk. Patients with a history of biliodigestive anastomosis or papillotomy, as well as those with intrahepatic cholangiocarcinoma or bile duct involvement, are at increased risk for post-interventional cholangitis or liver abscess formation [1]. In such patients, peri-interventional antibiotic prophylaxis (e.g. ceftriaxone, adjusted to local resistance profiles) is strongly advised.

#### 2.1.2 Hepatocellular carcinoma

Curative options include liver transplantation, surgical resection and thermal ablation, while liver-directed therapies such as TACE, transarterial embolization (TAE), TARE or SBRT provide locoregional control. For patients with cirrhosis and unresectable early-stage HCC, liver transplantation remains a curative option. Due to limited organ availability, bridging or downstaging strategies are often required, and brachytherapy may serve as an alternative in selected patients.

Patients with very early or early-stage HCC according to the Barcelona Clinic Liver Cancer (BCLC) classification benefit most from local ablative treatments [15]. Radiofrequency ablation is commonly used for small tumours but may be limited by tumour size or location. In these cases, interstitial HDR brachytherapy represents a minimally invasive alternative to thermal ablation. Unlike thermal ablation, interstitial HDR brachytherapy is not affected by heat-sink effects and can be used for lesions beyond conventional thermal-ablation limits in terms of size and location [16], [17], [18], [19], [20], [21], [22], [23].

#### 2.1.3 Cholangiocellular carcinoma

Surgical resection remains the only curative treatment for intrahepatic cholangiocarcinoma [24], [25]. In unresectable disease, locoregional treatments such as thermal ablation, SBRT or HDR brachytherapy may improve local control and delay intrahepatic progression. Brachytherapy may also be considered for larger tumours or as salvage treatment after recurrence [26], [27].

#### 2.1.4 Oligometastatic disease

The concept of oligometastatic disease is supported by randomised trials demonstrating improved outcomes with local metastasis-directed therapy in addition to systemic treatment [28], [29]. For unresectable liver metastases, multiple local ablative treatments are available. Radiotherapy approaches include SBRT and interstitial HDR brachytherapy. While SBRT is widely used, brachytherapy can deliver high local doses with a favourable safety profile [3], [5]. In selected patients, interstitial HDR brachytherapy may also be considered as consolidation or cytoreductive local treatment after systemic therapy [30], [31], [32].

### 2.2 Frail and elderly patients

With increasing life expectancy and rising cancer incidence, the proportion of elderly and frail patients with primary or secondary liver malignancies is expected to grow. In this population, interstitial HDR liver brachytherapy offers a compelling treatment option due to its ability to deliver highly conformal ablative doses with minimal impact on surrounding normal tissues [33].

Older patients often present with multiple comorbidities, functional limitations, and psychosocial challenges that complicate the administration of systemic therapies or extensive surgical interventions. The non-systemic nature of brachytherapy and its favourable safety profile make it an attractive option in this setting. Careful patient selection is essential, with key considerations including performance status, comorbidity burden, liver function, and life expectancy. Poor performance status (ECOG ≥3) or advanced hepatic dysfunction (Child-Pugh Class C or higher) may represent contraindications to treatment [22].

Although dedicated prospective studies focusing specifically on the frail elderly population are lacking, the minimally invasive nature and reported safety profile of liver HDR brachytherapy may make it a suitable option in selected patients [16]. Importantly, the technique is associated with low rates of severe morbidity and complications, reducing the risk of unplanned hospitalization and supporting its use in both curative and palliative clinical pathways [22].

Given the tendency toward undertreatment in elderly cancer patients—a factor known to contribute to decreased survival—brachytherapy represents a well-tolerated and effective modality that may help to bridge this treatment gap [34]. Nonetheless, further dedicated research is required to more precisely define the benefit-risk profile of interstitial HDR brachytherapy in frail elderly patients and to establish optimal treatment protocols for this growing patient subgroup.

## 3 Technique and workflow of interstitial HDR liver brachytherapy

### 3.1 Image guidance for catheter implantation

#### 3.1.1 CT Guidance

Non-contrast or contrast-enhanced CT scans of the liver are routinely used for both pre-procedural implant planning and real-time image guidance during catheter implantation (see Fig. 1). Fluoroscopic CT is particularly useful, as it allows dynamic adaptation to respiratory motion through voluntary breath-holds, which can significantly impact target positioning and catheter placement accuracy [35].

**Fig. 1:**
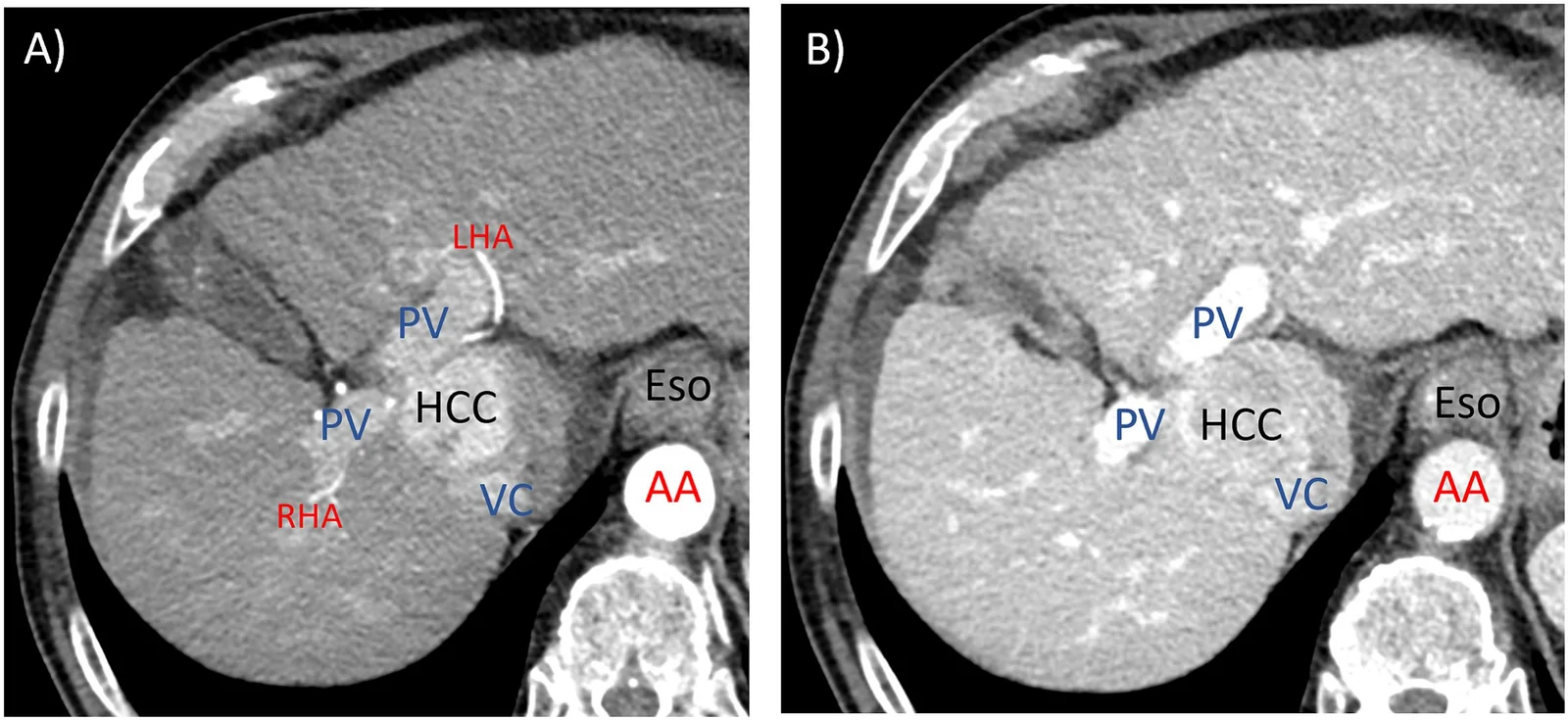
Liver anatomy and vascular structures. The hepatocellular carcinoma (HCC) typically shows (A) arterial phase hyperenhancement with (B) subsequent portal and delayed phase washout. AA: Abdominal Aorta, RHA: Right hepatic artery, LHA: Left hepatic artery, PV: Portal Vein, VC: Vena cava, Eso: Oesophagus, HCC: Hepatocellular carcinoma.

#### 3.1.2 Ultrasound guidance

Ultrasound is a valuable alternative imaging modality. It offers real-time imaging without ionizing radiation and allows for flexible probe positioning to visualize superficial or peripherally located lesions. Ultrasound can improve procedural efficiency by facilitating rapid and accurate access to the target area and enables continuous monitoring of vascular structures during needle advancement [36].

#### 3.1.3 MRI guidance

For small or poorly visualized lesions on CT, interventional MRI may offer superior soft tissue contrast and enhanced visualisation of the target and surrounding anatomy (see Fig. 2). The use of hepatocyte-specific contrast agents can further improve lesion visualisation, with contrast enhancement typically lasting up to 90 min. MRI fluoroscopy enables real-time needle tracking and flexible selection of slice orientation, allowing accurate catheter placement even in complex anatomical regions. MR-safe needles and plastic brachytherapy catheters ensure procedural compatibility and safety within the MRI environment [37], [38].

**Fig. 2:**
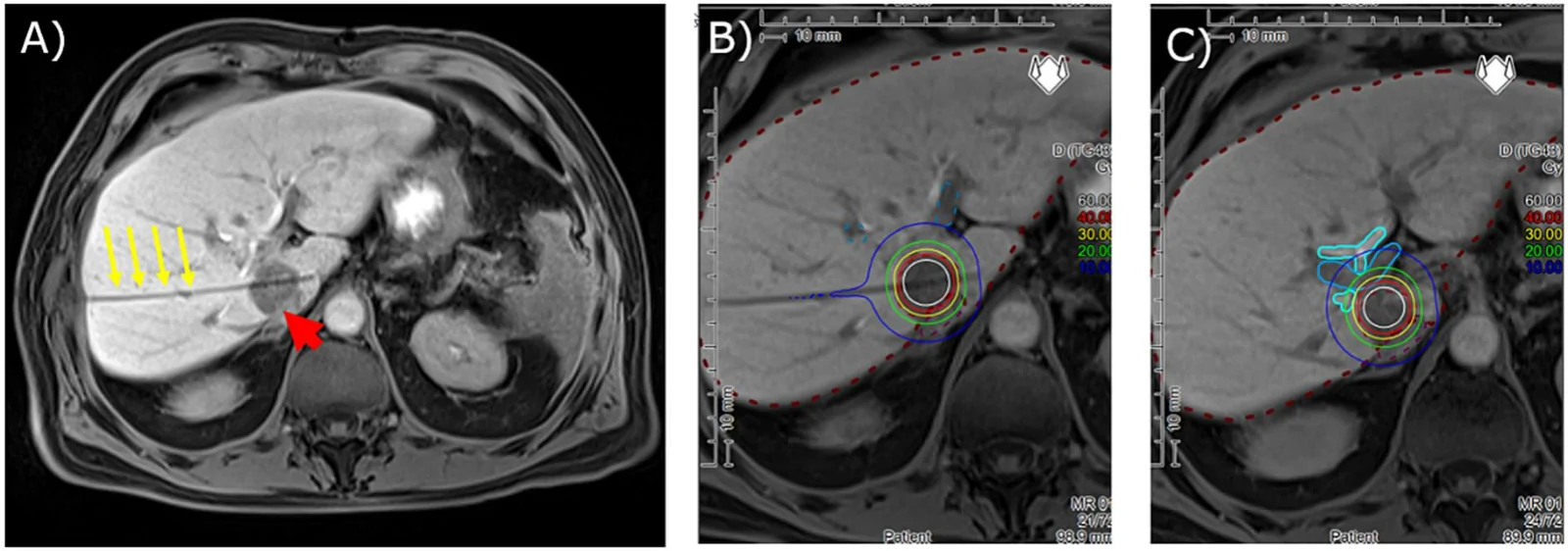
MR-guided brachytherapy. (A) Contrast-enhanced MRI. The brachytherapy catheter (yellow arrows) is placed in the metastasis (red arrow). (B) and (C) show the dose distribution optimized to 1 × 20 Gy (green isoline). In this example, the puncture tract was treated with ~5 Gy to the catheter surface (see B). Blue isoline: 10 Gy, green isoline: 20 Gy, yellow isoline: 30 Gy, red isoline: 40 Gy, white isoline: 60 Gy. Organs at risk include the portal vein (blue) and the central bile ducts (light blue). (For interpretation of the references to colour in this figure legend, the reader is referred to the web version of this article.)

### 3.2 Implantation techniques

Interstitial HDR brachytherapy for liver tumours can be performed using different implantation techniques, depending on institutional expertise, tumour characteristics, and available imaging modalities. The most commonly employed approaches include the direct implantation technique and the Seldinger technique, both performed under image guidance—typically using CT or MRI with ultrasound used in selected cases. Each method has specific advantages and considerations.

In order to have an optimal display of the disease burden and for therapy planning, recent staging imaging must be available. In addition to multiphase thoracoabdominal CT, a liver MRI with hepatocyte-specific contrast media is recommended. Studies support the additive value of liver MRI with hepatocyte specific contrast media in therapy decision making [39], [40], [41]. For complex lesions (large in size or complex geometry), especially when the lesion is intended to be treated in multiple sessions, pre-planning using the staging imaging can be helpful to simulate the treatment and to have a better understanding for optimal catheter positioning. In selected patients, an angiographic occlusion balloon can be placed between the gastric wall and the liver to physically displace the stomach and reduce the radiation dose [42].

The procedure can be performed under general anaesthesia. However, local anaesthesia or conscious sedation may be safely used in appropriately selected patients when administered by trained personnel under standardised monitoring and with immediate anaesthesiological support available [43]. Standard premedication typically includes intravenous administration of an antiemetic to prevent nausea and vomiting. Optionally, also an antifibrinolytic agent to minimise bleeding, as well as antibiotics to reduce the risk of infection can be applied. These medications are usually administered approximately 30 min prior to the intervention.

#### 3.2.1 Direct implantation technique

The direct implantation approach is commonly employed, particularly in centres where radiation oncologists perform the catheter placement [30], [31], [32]. While this approach is theoretically feasible for lesions in all hepatic segments, its practical applicability must be evaluated on a case-by-case basis, considering patient anatomy, tumour position, and the technical difficulty of the access route (see Fig. 3). Implantation may be technically challenging in tumour locations without direct tumour access which may require the creation of an angulated puncture tract.

**Fig. 3:**
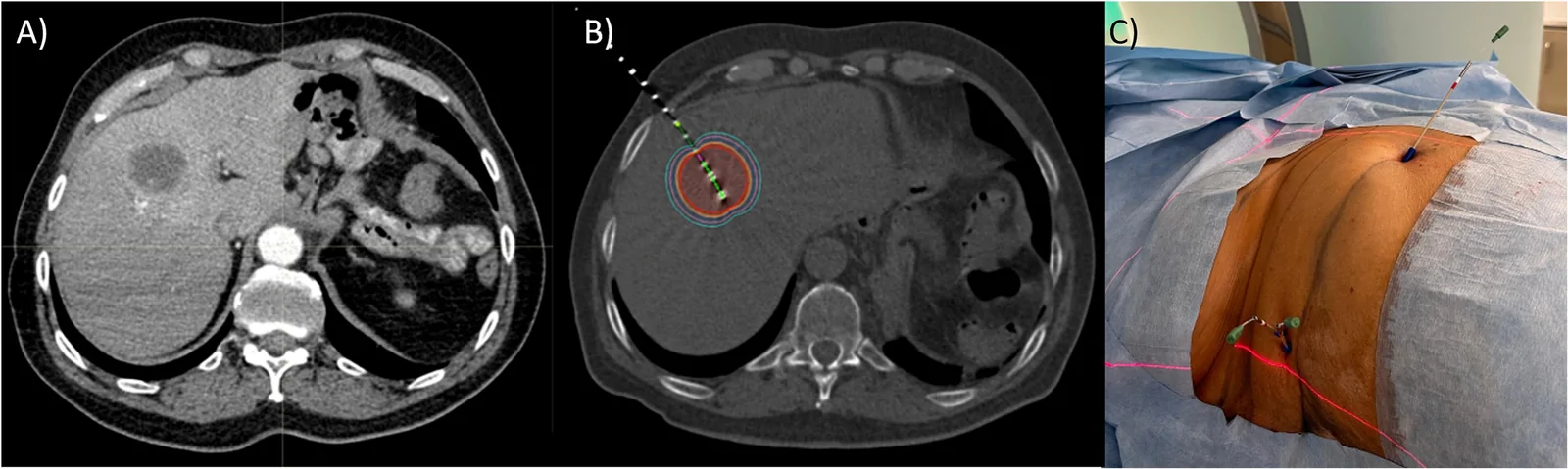
An example for CT-guided catheter implantation using the direct puncture technique. (A) contrast-enhanced CT showing the liver lesion. (B) treatment-planning CT after catheter placement, with the brachytherapy catheter visualized using a dummy marker and dose distribution. (C) clinical setup with the percutaneously inserted liver brachytherapy catheter in situ.

Contrast-enhanced CT imaging is used to precisely localise the tumour and visualize adjacent vascular structures, ensuring that the planned needle trajectory avoids any vessels, before marking the entry points on the skin surface. A small skin incision is made and the catheter is introduced through the intercostal space. Real-time CT fluoroscopy is used throughout the intervention to guide catheter placement. Brachytherapy catheters (6F) with lengths of 240 mm or 320 mm and containing an internal obturator/stylet to provide sufficient stiffness during insertion are advanced under continuous CT fluoroscopy image guidance until the tip is securely positioned. Due to the offset of the radioactive source within the catheter, the catheter tip should extend slightly beyond the tumour margin. Once in place, catheters must be securely fixed to the skin to ensure positional stability and enable precise dose delivery during treatment (see Fig. 3). After treatment, catheters are simply removed, as there is no option for puncture tract sealing.

#### 3.2.2 Seldinger technique

The Seldinger technique represents an alternative approach to catheter implantation and may be preferred in centres with established interventional radiology expertise [44]. The procedure begins with the placement of a midsized hollow puncture needle (17 or 18G) under image guidance. Once the needle position is confirmed, a stiff guidewire (e.g. Amplatz Super Stiff) is inserted through the needle. The needle is then removed and replaced with a hydrophilic coated 6F angiographic sheath (e.g. Radiofocus Introducer II), which is advanced over the guidewire. Following successful sheath placement, the guidewire is withdrawn, and a flexible 6F brachytherapy catheter (e.g. Primed Halberstadt) is advanced to the desired position within the lesion in the liver. Once the catheter is appropriately placed, the sheath is secured to the skin using sutures to ensure stability throughout the treatment (see Fig. 4). Upon completion of therapy, the catheter is removed and the puncture tract is sealed using absorbable haemostatic material (e.g., gelfoam plugs) to reduce the risk of post-procedural bleeding [16], [45]. If histological confirmation is required, a biopsy may be obtained through the same puncture tract before catheter placement.

**Fig. 4:**
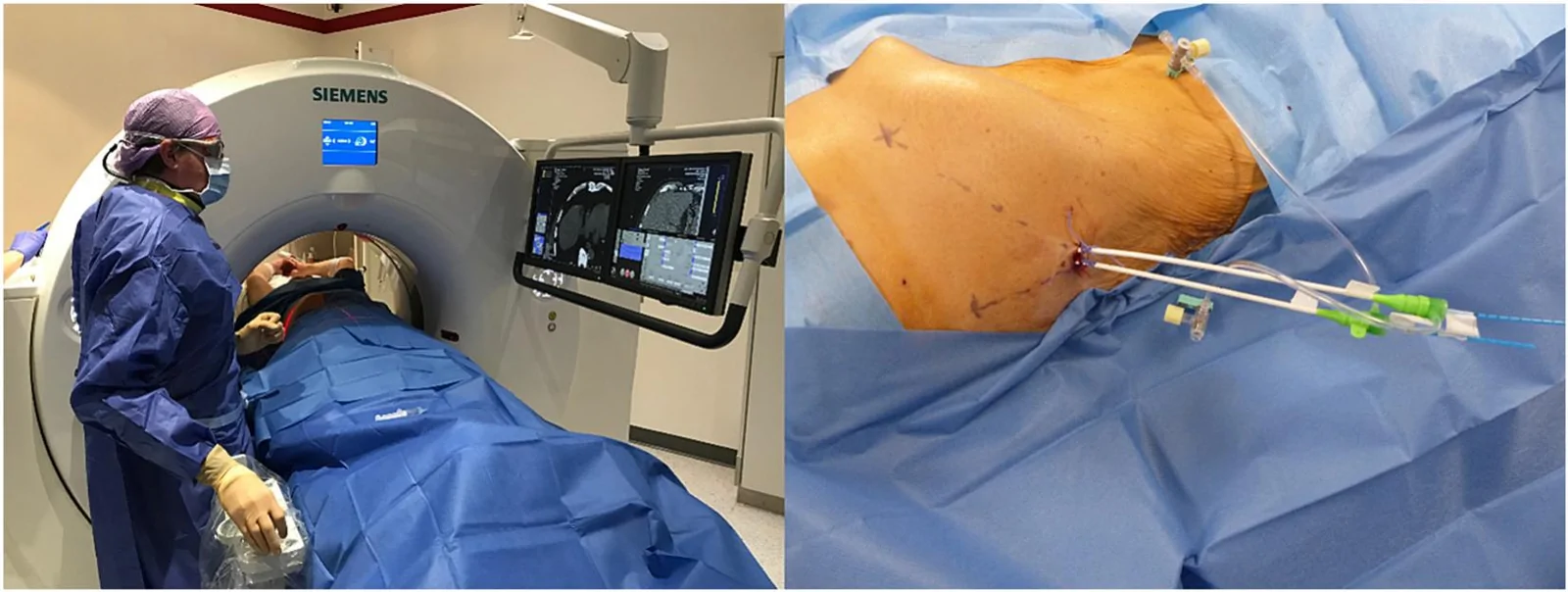
An example of the set-up (left image) used for CT-guided catheter implantation using the Seldinger technique. The blue brachytherapy catheter (right image) can be seen inside the green/white. angiography sheath. The transfer tube of the afterloader (not included in the image) will be connected directly to the blue brachytherapy catheter. (For interpretation of the references to colour in this figure legend, the reader is referred to the web version of this article.)

This technique offers several practical advantages compared to the direct implantation technique. The initial 17- or 18G puncture needle is thinner than the 6F catheter and can reduce imaging artefacts during initial access. In addition, the smaller size of the sharp interventional needles may help to minimise tissue trauma. An additional advantage of the Seldinger technique is its compatibility with MRI, which can be particularly beneficial when treating small or difficult-to-visualize lesions. Furthermore, the technique facilitates reliable sealing of the catheter tract following treatment, contributing to a reduced risk of post-procedural bleeding. As with the direct technique, in appropriately selected patients, the procedure can usually be performed without the need for general anaesthesia.

### 3.3 Treatment planning

#### 3.3.1 Imaging and contouring

Following catheter implantation, a high-resolution post-procedural CT scan with a slice thickness of 1–3 mm of the entire liver is obtained to facilitate treatment planning. Target volume delineation including the gross tumour volume (GTV) and clinical target volume (CTV), plus OoI is complemented by additional imaging modalities such as diagnostic CT, MRI, or PET/CT. The process of image fusion can present technical challenges, particularly due to differences in breath-hold levels and anatomical changes in liver morphology, which may affect the accuracy of image registration. To support reliable alignment between planning and diagnostic datasets, advanced image registration tools (e.g. deformable fusion) can be helpful. The GTV comprises the visible tumour. The CTV includes the GTV with an additional margin to account for potential microscopic extension. Although no consensus margin has been established for interstitial liver brachytherapy, margins of 0–5 mm are used depending on institutional protocols or specific clinical considerations. An additional planning target volume (PTV) margin is typically not applied (CTV=PTV).

When catheter implantation is performed under interventional MRI guidance, an additional CT scan may be required for treatment planning. However, treatment planning can also be performed directly on MRI, provided that adequate catheter visualisation and geometric accuracy are ensured. Particular attention should be paid to MRI-specific limitations, including image distortion and susceptibility artefacts, especially near the liver dome or adjacent to air–tissue interfaces. On CT, the catheter tip may be identified using a radiopaque CT dummy inserted into the catheter, an integrated radiopaque marker present in the tip some brachytherapy catheter types, or the air-filled catheter lumen. On MRI, catheter reconstruction may similarly rely on the air-filled lumen or on dedicated MR-compatible dummy wires or marker inserts [46]. The geometric offset between the visualized catheter tip or marker and the first active source dwell position must be accounted for according to the specific catheter system and its clinical commissioning.

Where available, the use of structure atlases and AI-assisted contouring tools can facilitate efficient delineation of OoI, although final review by a clinician remains essential [47]. The selection and number of structures to be contoured should follow institutional protocols. However, all OoI potentially exposed to relevant dose levels (see Table 1) must be appropriately delineated.

**Table 1:** ESTRO consensus recommendations for dose reporting.

Standard reporting parameters		
Structure	Parameter	Description/Goal
Target volume	D100%	Prescribed dose covering 100% of the reported target volume
Target volume	D98%	Prescribed dose covering 98% of the reported target volume
Organs of interest (OoI)	D 0.1 cm^3^	Dose to 0.1 cm^3^ of the OoI volume
OoI	D 1.0 cm^3^	Dose to 1cm^3^ of the OoI volume
Parallel Organs (liver/kidney)	V 10 Gy	Volume receiving 10 Gy or more
Parallel Organs (liver/kidney)	V 5 Gy	Volume receiving 5 Gy or more
**Advanced reporting parameters**		
Target volume	D95%	Prescribed dose covering 95% of the reported target volume
Target volume	D90%	Prescribed dose covering 90% of the reported target volume
Target volume	V150%	Volume receiving ≥150% of the prescription dose
Target volume	V200%	Volume receiving ≥200% of the prescription dose
Target volume	V300%	Volume receiving ≥300% of the prescription dose
Quality Indices	CI (Conformity)	Ratio of prescription isodose volume to target volume
Quality Indices	DHI (Homogeneity)	Dose Homogeneity Index

#### 3.3.2 Treatment planning

Treatment planning should be performed using a clinically commissioned brachytherapy treatment planning system (TPS) that complies with applicable regional regulatory requirements. Dose optimization can be conducted manually or supported by automated tools, depending on system capabilities and user preference. Dose calculation typically follows the TG-43 formalism, which assumes a water-equivalent medium. If model-based dose calculation is used, implementation should follow TG-186 recommendations and account for uncertainties in material assignment; liver-specific validation data are available for CT-based HDR brachytherapy [48], [49].

In interstitial HDR liver brachytherapy, treatment is most commonly delivered in a single fraction. The prescribed dose is determined based on tumour histology, as outlined in Table 2, and aims to achieve full coverage of the reported target volume (GTV or CTV). Ideally, the prescription dose should encompass the entire reported target volume, reaching D100%, which indicates that the minimum dose delivered covers 100% of the reported target volume (see example in Fig. 5). To assess dose coverage, key parameters—D100%, D98%, D95%, and D90%—should be reported alongside the median target-volume dose. These metrics enable a robust evaluation of dose conformity and target coverage.

**Table 2:** ESTRO consensus recommendations for dose prescription and organs of interest (OoI) constraints. Suggested constraints are based on available interstitial liver HDR BT data, published single-fraction tolerance data, and expert consensus. [1], [12], [44], [50], [51], [52], [53].

Target volume Planning aim	D 100%	Organs of Interest constraints	D 0.1 cm^3^	D 1.0 cm^3^
Metastases of colorectal cancer	25 Gy	Oesophagus	15 Gy	12 Gy
Cholangiocellular Carcinoma	20 Gy	Stomach	15 Gy	12 Gy
Hepatocellular Carcinoma	15 Gy	Duodenum	15 Gy	12 Gy
Other metastases	20 Gy	Small bowel	15 Gy	12 Gy
		Large bowel	15 Gy	12 Gy
		Heart	22 Gy	
		Great vessels (Aorta, Vena cava inferior)	37 Gy	
		Gall bladder	20 Gy	
		Spinal canal	12 Gy	10 Gy
		Skin surface	10 Gy	
		Main bile duct	20 Gy	18 Gy
		Liver	V10Gy < 33% of healthy liver volume	
		Liver	V5Gy < 66% of healthy liver volume

**Fig. 5:**
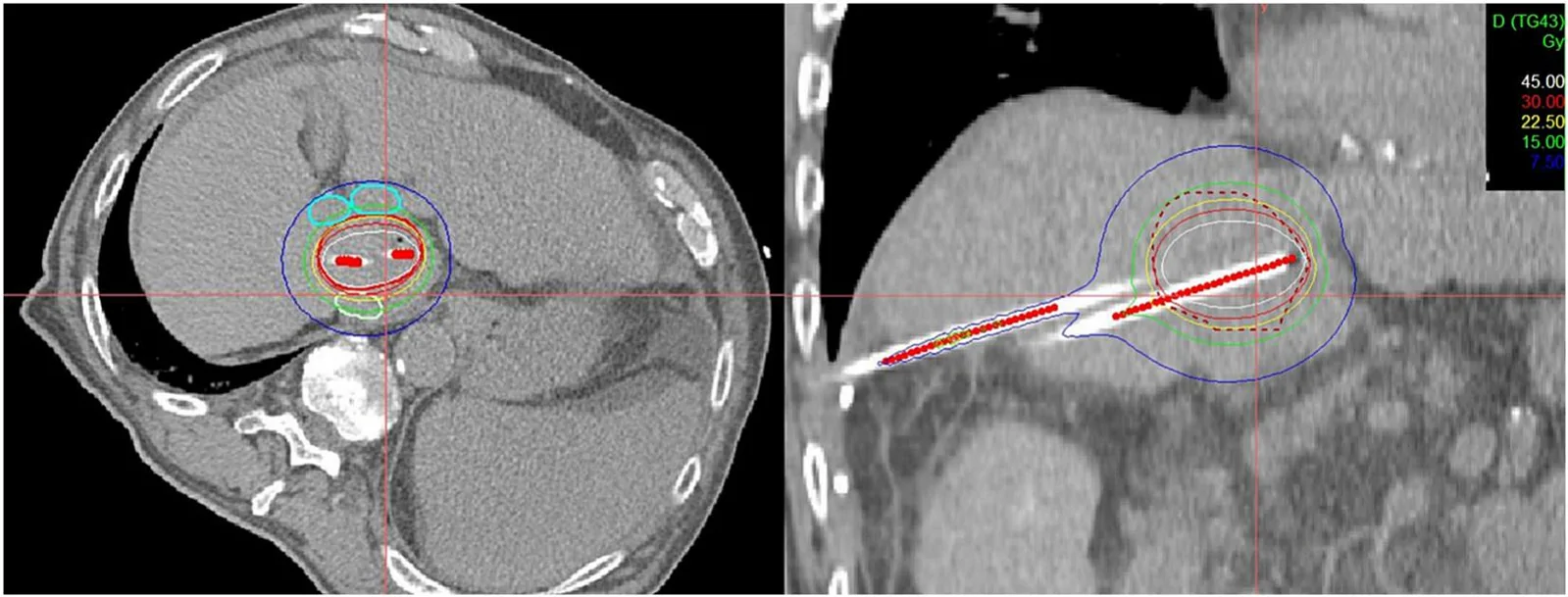
Treatment plan of HCC in Segment I (red contour) with dose optimized to 1 × 15 Gy (green isoline). In this example, the puncture tract is treated with ~5 Gy to the catheter surface (see right image). Blue isoline: 7.5 Gy, green isoline: 15 Gy, yellow isoline: 22.5 Gy, red isoline: 30 Gy, white isoline: 45 Gy. (For interpretation of the references to colour in this figure legend, the reader is referred to the web version of this article.)

Dose reporting should follow the principles of ICRU Report 83 (for intensity-modulated radiation therapy (IMRT)) [54] and ICRU Report 89 (for cervix brachytherapy) [55], both of which are largely applicable in interstitial HDR liver brachytherapy. Modern HDR brachytherapy requires Level 2 dose reporting, including detailed DVH metrics, technical planning data, and documentation of the TPS configuration. While the D2% value may be of limited relevance due to the steep dose gradients around the source, reporting of high-dose volumes such as V150%, V200%, and V300% is encouraged to reflect dose heterogeneity within the target (see Table 1). The median dose (D50%) to the target volume is generally preferred over the mean dose, as the latter can be disproportionately affected by small regions of extremely high dose, which commonly occur due to the steep dose gradients around the source.

For OoI, dose constraints depend on the type of organ. For serial structures (e.g., oesophagus, stomach, duodenum, bowel, heart, bile ducts, spinal canal), constraints are generally defined as D0.1 cm^3^ or D1.0 cm^3^, indicating the minimum dose received by the most exposed x cm^3^ of tissue. For parallel organs (e.g., liver parenchyma, kidneys), mean dose and volumetric thresholds (e.g., V10Gy, V15Gy) are more appropriate. Suggested OoI dose constraints are provided in Table 2. When balancing target coverage and OAR sparing, adherence to established OoI dose constraints must always be given priority, even if this results in a compromise of target dose coverage. The puncture tract may be irradiated with approximately 5 Gy to minimise the risk of seeding.

The typical source strength of a freshly loaded Iridium-192 source is approximately 40 kU (corresponding to an activity of ~370 GBq), resulting in a dose rate exceeding 7 Gy per minute at 1 cm distance in water. These dose rates lead to radiobiological effects comparable to single-fraction or strongly hypofractionated EBRT techniques, such as SBRT or stereotactic radiosurgery (SRS).

Finalised treatment plans must undergo thorough review and quality assurance prior to approval and treatment delivery. In centres where the treatment planning system is provided by a different vendor than the afterloading device, particular attention must be paid to ensure accurate transfer of planning parameters and avoid potential inconsistencies. Routine verification of the source strength is required on a daily basis, and regular constancy checks of the treatment planning system must be conducted in accordance with local quality assurance standards.

## 4 Oncological outcomes

### 4.1 Primary liver tumours: Hepatocellular carcinoma and cholangiocarcinoma

The available evidence is dominated by retrospective single-centre series; prospective and comparative studies remain limited. Most published studies focus primarily on local tumour control and use heterogeneous patient selection and follow-up schedules [26].

Between 2004 and 2021, outcomes from 18 studies comprising a total of 618 patients treated with single-fraction interstitial HDR brachytherapy for primary or secondary liver tumours have been reported [26]. Among these, HCC and CCC represent the most common primary malignancies of the liver.

#### 4.1.1 Hepatocellular carcinoma

Six studies published between 2010 and 2019 examined interstitial HDR brachytherapy in a total of 284 patients with HCC (see Table 3) [16], [17], [18], [19], [20], [21]. Median follow-up ranged from 9.6 to 41.2 months. Despite heterogeneous patient cohorts and varying pre-treatments—including systemic therapies, local ablation, or transarterial approaches—local control (LC) rates were consistently high. Reported LC ranged from 95.9% at 6 months to 91.5% at a median follow-up of 20 months. Notably, Collettini et al. evaluated large (5–7 cm) and very large (>7 cm) HCCs and reported a local control rate of 93.3% at a median follow-up of 12.8 months, supporting the feasibility of HDR brachytherapy even for tumours beyond conventional size limits for thermal ablation [17]. More recently, Ümütlü et al. reported favourable local tumour control in HCC lesions treated with interstitial HDR brachytherapy both within and beyond conventional thermal-ablation limits, with 12-, 24-, and 36-month local tumour control rates of 87%, 75%, and 73%, respectively [23].

**Table 3:** Results of interstitial liver brachytherapy in HCC.

Entity	Study (year)	Study design	Patients (% male)	Treated lesions (number)	Lesion size (range)	Median follow-up (months)	D_100%_ (range)	Local control (months)	Median overall survival
Hepatocellular carcinoma (HCC)	Mohnike (2010) [16]	Prospective, single-centre	83 (79.5%)	140	4.4 cm (1–15 cm)	9.6 m	15 Gy (12–15 Gy)	95% at 12 m	19.4 m 64% at 12 m 25% at 36 m
	Collettini (2012) [17]	Retrospective, single-centre	35 (83%)	35	7.1 cm (5–12 cm)	12.8 m	15.8 Gy (15–20 Gy)	93.3% at 12.8 m	15.4 m
	Collettini (2015) [18]	Retrospective, single-centre	98 (83.7%)	192	5 cm (1.8–12 cm)	20 m	16.5 Gy	91.5% at 20 m	29.2 m 87.6% at 12 m 57.3% at 24 m 41.6% at 36 m
	Schnapauff (2015) [21]	Retrospective, single-centre	19 (84.2%)	48	2.5 cm (0.4–5.7 cm)	33 m	20 Gy	95.9% at 6 m	50 m
	Denecke (2015) [19]	Retrospective, single-centre	12 (75%)	12	3.6 cm	n/a	18.9 Gy (15–25 Gy)	90% at 12 m 90% at 36 m	n/a
	Mohnike (2019) [20]	Randomised phase II, single-centre	37 (84%)	n/a	4.5 cm (3–6.5 cm)	41.2 m	15 Gy	Time to untreated progression: 67.5% at 12 m	78.4% at 12 m
	Xu (2021) [56]	Retrospective, single-centre	114 (78.9%)	142	3.6 cm (± 2.3 cm)	24 m	20 Gy	median local PFS 36.8 m median time to local progression 50.1 m	not reached 79.8% at 12 m 50% at 24 m
	Ümütlü (2024) [23]	Retrospective, single-centre	146 (81.5%)	216	2.9 cm (0.6–11.1 cm)	16 m	15 Gy	87% at 12 m 75% at 24 m 73% at 36 m	not reached 70% at 36 m

Overall survival (OS) at 12 months ranged between 64% and 87.6% [16], [18], again reflecting variability in patient selection, tumour burden, and the availability of systemic treatment options. At 36 months, OS was reported between 25% and 41.6%. These data support interstitial HDR brachytherapy as an effective local treatment in patients with HCC, particularly those with limited systemic options or contraindications to other local therapies.

Of particular interest is the role of interstitial HDR brachytherapy as a bridging therapy to liver transplantation. Denecke et al. [19] compared interstitial HDR brachytherapy with the standard bridging modality of TACE. Their findings suggest that interstitial HDR brachytherapy provides comparable or even superior tumour response and post-transplant recurrence rates, indicating its utility in patients particularly for those who are ineligible for or non-responders to TACE. More recently, histopathological analysis of explanted livers after HDR brachytherapy demonstrated complete pathological necrosis in 65.5% of treated lesions, with all remaining lesions showing >50% necrosis [57].

#### 4.1.2 Intrahepatic cholangiocarcinoma

Two studies specifically evaluated the role of interstitial brachytherapy in patients with unresectable intrahepatic CCC (Table 4). Schnapauff et al. reported on 15 patients, achieving a median local control (LC) duration of 10 months and a median overall survival of 14 months at a median follow-up of 18 months [27]. In a larger cohort, Jonczyk et al. analysed 61 patients and investigated outcomes stratified by tumour size [58]. At 12 months, LC was 87% for lesions smaller than 4 cm and 78% for those larger than 4 cm. Although no statistically significant difference in overall survival (OS) was observed between the two groups, median OS was 15.5 months for smaller lesions versus 10.0 months for larger tumours.

**Table 4:** Results of interstitial liver brachytherapy in CCC.

Entity	Study (year)	Study design	Patients (% male)	Treated lesions (number)	Lesion size (range)	Median follow-up (months)	D_100%_ (range)	Local control (months)	Median overall survival
Cholangiocellular carcinoma (CCC)	Schnapauff (2012) [27]	Retrospective, single-centre	15 (33%)	22	5.2 cm (1–18 cm)	18 m	20 Gy (15–20 Gy)	10 m (1–25 m)	14 m
	Jonczyk (2018) [58]	Retrospective, single-centre	61 (62%)	142	2 cm (1–3.8 cm) versus 6.9 cm (4–14.8 cm)	n/a	20 Gy (15–20 Gy)	87% (<4 cm) versus 78% (>4 cm) at 12 m	15.5 m (<4 cm) versus 10 m (>4 cm)

Both studies reflect real-world clinical settings with complex patient profiles, often following extensive prior therapies. These findings support interstitial HDR brachytherapy as a feasible and effective local treatment for CCC, particularly in patients with limited therapeutic alternatives or tumour progression despite systemic or regional treatment.

In summary, current evidence suggests that interstitial HDR brachytherapy offers a valuable treatment option for primary liver cancers, including HCC and CCC, achieving durable local control and acceptable survival outcomes even in challenging patient populations. Further prospective studies may help clarify its role in the treatment algorithm, particularly in comparison to or in combination with other loco-regional therapies.

### 4.2 Liver metastases from colorectal cancer

Liver brachytherapy for colorectal cancer (CRC) metastases has been practiced in Europe for almost three decades [22], generating a substantial body of clinical evidence (see Table 5). One of the earliest prospective analyses was performed by Ricke et al., who treated 37 patients with CT-guided interstitial HDR brachytherapy, for liver tumours ranging in size from 2.5 to 11 cm (mean: 4.8 cm). Of these, 27 lesions were of colorectal origin. LC was achieved in 87% of patients treated with interstitial HDR brachytherapy at nine months. OS at 12 months was 69% [35].

**Table 5:** Results of interstitial liver brachytherapy in patients with colorectal cancer (CRC).

Entity	Study (year)	Study design	Patients with CRC	Treated lesions (number)	Lesion size (range)	Median follow-up (months)	D_100%_ (range)	Local control	Overall survival
Colorectal Cancer	Ricke (2004) [35]	Phase I-II Single-Center	27/37	N/A	4.8 cm (2.5–11 cm)	14m	17 Gy (10–20 Gy)	9m: 87%	12m: 69%
	Ricke (2004) [59]	Phase II Single-Center	12/20	N/A	7.7 cm (5.5–10.8 cm)	13m	17Gy (12–25 Gy)	6m: 80% 9m: 53%	12m: 83%
	Tselis (2013) [14]	Retrospective Single-Center	14/41	19	84 ml (38–1348 ml)	12.4m	20 Gy (7–32 Gy)	6m: 89% 12m: 73% 18 m: 63%	N/A
	Collettini (2014) [60]	Retrospective Single-Center	80/80	179	2.8 cm (0.8–10.7 cm)	16.9m	19.1 Gy (15–20Gy)	12m: 88.3% 24 m: 81.2% 36m: 68.4%	12m: 87.6% 24 m: 57.3% 36m: 41.6%
	Kieszko (2018) [11]	Retrospective Single-Center	46/61	N/A	42.9 ml (2.7–174.9 ml)	11m	Mean D90: 20.2 Gy (15–25Gy)	6m: 88.7% 12m: 70.7%	6m: 96.7% 12m: 79.6%
	Walter (2021) [44]	Retrospective Single-Center	54/106	71	3.2 ± 1.9 cm	9m	19.3 ± 4.8 Gy	6m: 84.1% 12m: 50.6%	6m: 95.2% 12m: 72.4%
	Xu (2021) [56]	Retrospective, single-centre	50/164	81	4.0 cm (± 2.4 cm)	13.8 m	20 Gy	median local PFS 12.4 m median time to local progression 9.9 m	78% at 6 m 50% at 12 m

In a subsequent prospective, non-randomised phase II trial, Ricke et al. evaluated 20 patients with large (up to 10 cm) or unfavourably located primary or secondary liver tumours not amenable to thermal ablation. Twelve patients had CRC as the primary tumour. They reported LC rates of 80% and 53% at 6 and 9 months, respectively, with an OS of 83% at 12 months [59]. Further investigation by the same group revealed a clear dose-response relationship: while overall LC at 15 months was 75%, it increased to 95% when doses were > 25 Gy, underscoring the importance of adequate dose escalation for effective tumour control [45]. In a comparative analysis, Xu et al. reported a shorter median local progression-free survival for colorectal liver metastases than for HCC following interstitial HDR brachytherapy (12.4 vs. 36.8 months) [56].

Additional studies have corroborated these findings. Tselis et al. treated 41 patients with a total of 50 unresectable liver tumours, including 14 patients with CRC metastases. Their results showed LC rates of 89%, 73%, and 63% at 6, 12, and 18 months, respectively, for metastatic lesions [61]. Collettini et al. provided long-term data from 80 patients with 179 colorectal liver metastases treated in either single or multiple fractions. They reported LC rates of 88.3%, 81.2%, and 68.4% at 12, 24, and 36 months, respectively, with corresponding OS rates of 87.6%, 57.3%, and 41.6% [60].

Kieszko et al. evaluated outcomes in 61 patients with 73 secondary liver tumours, of which 46 were CRC derived. At 6 months, LC and OS rates were 88.7% and 96.7%, respectively, as well as 70.7% and 79.6% at 12 months [11]. Similarly, Walter et al. reported on a larger cohort of 106 patients with 244 liver metastases treated with interstitial HDR brachytherapy in an oligometastatic setting (up to five lesions), including 54 patients with CRC. OS was 92.3% at 6 months and 76.3% at 12 months. Stratified data showed CRC-specific OS rates of 95.2% and 72.4% at the same time points. Local recurrence-free survival (LRFS) for the whole group was 88.4% at 6 months and 71.5% at one year, with CRC patients showing lower LRFS (84.1% and 50.6%) compared to those with mixed histology (92.4% and 92.4%). Progression-free survival (PFS) across all patients was modest—36.8% at 6 months and 15.5% at 12 months—with CRC patients demonstrating slightly lower PFS than those with other primaries. Importantly, subgroup analysis revealed that CRC patients who received a D100% of ≥20 Gy in all treated lesions and fractions had significantly improved LRFS [44].

A retrospective matched-pair analysis by Friedrich et al. explored the impact of combination therapy. Forty-four patients with unresectable CRC liver metastases were treated with interstitial HDR brachytherapy either alone or in combination with TACE using irinotecan-loaded microspheres. The combination approach led to longer median PFS (5 vs. 2 months), and substantially lower rates of local (23% vs. 68%) and intrahepatic progression (50% vs. 95%) compared to interstitial HDR brachytherapy alone. A trend toward longer LC (17 vs. 9 months) was also observed following combination therapy, suggesting potential synergy between local radiotherapy and intra-arterial chemotherapy [62].

### 4.3 Liver metastases from other solid tumours

Among 27 publications reporting clinical outcomes of interstitial HDR brachytherapy for secondary liver malignancies of non-colorectal origin (Table 6), 5 were prospective studies and 22 were retrospective in design. Seventeen publications focused on single primary tumour entities—most notably breast cancer (*n* = 5) [63], [64], [65], [66], [67], renal cell carcinoma (*n* = 2) [68], [69], pancreatic cancer (*n* = 2) [70], [71], and gastric or gastroesophageal junction tumours (*n* = 2) [72], [73]. Ten additional studies presented pooled analyses across multiple tumour types. Where data were distinguishable, outcomes from 850 patients with a total of at least 1573 liver metastases were evaluated. Tumour sizes varied considerably, with treated metastases ranging from 0.4 cm to 16 cm in diameter, and volumes between 36.7 cm^3^ and 84 cm^3^.

**Table 6:** Results of interstitial liver brachytherapy in non-hepatic/non-colorectal primaries.

Entity	Study (year)	Study design	Patients (% male)	Treated lesions (number)	Lesion size (range)	D_100%_ (range)	Local control [month]	Median Overall Survival (range)
Breast cancer	Collettini (2012) [63]	Retrospective, single-centre	37 (0)	80	2.6 cm (0.8–7.4 cm)	18.6 Gy (±2.27 Gy)	94.6% at 12 m	18 m (3–39 m)
	Wieners (2011) [64]	Prospective phase II, single-centre	41(0)	115	4.4cm (1.0–11 cm)	18.5 Gy (12–25 Gy)	97% at 6 m 93.5% at 18 m	n/a
	Thormann (2022) [65]	Retrospective, single-center	60 (0)	n/a	2.9 cm (0.5–9.3 cm)	16.2 Gy (6.9–21.9 Gy)	n/a	27 m
	Seidensticker (2015) [66]	Retrospective, single-centre, mixed ablative techniques	59 (2)	68/33 x BT; 49% extrahep. Met.	4.1 cm (1.0–16 cm)	15 Gy	n/a	21.9 m
	Bilski (2026) [67]	Retrospective, multi-centre	107 (0)	170	5.0 cm (1.2–18.5 cm)	20 Gy (15–25 Gy)	46.2% at 24 m	37 m
Neuroendocrine tumours	Schippers (2017) [74]	Retrospective, single-centre	27 (48)	52	2.1 cm (0.7–11.0 cm)	15–20 Gy (target)	92% at 12 m 83% at 36 m	36 m (4.2–106.1 m)
Ovarian cancer	Colletini (2011) [75]	Retrospective, single-centre	7	12	3.1 cm (1.3–12 cm)	15 Gy	100% at avg. FU 15.4 m	100% at 12 m
Renal carcinoma	Geisel (2012) [68]	Retrospective, single-centre	10 (70)	16	3.8 cm (1–8.2 cm)	n/a	93.8% at avg. FU 21.6 m ± 13.7 m	n/a
	Omari (2019) [69]	Retrospective, single-centre	14 (64.3)	54	1.8 cm (0.5–13.9 cm)	16.1 Gy	92.6% avg. FU 10.2 (range 2.4–73.6 m)	51.2 m (1.0–27.8 m)
Gastrointestinal stroma tumours	Omari (2019) [76]	Retrospective, single-centre	10 (90)	40	2.4 cm (0.6–11.2 cm)	15 Gy (6.7–21.9 Gy)	97.5% at 25 m median FU	37.3 m (11.4–89.7 m)
Anal cancer	Heinze (2018) [77]	Retrospective, single-centre	7 (28.5)	38	1.2 cm (0.4–6.2 cm)	16.2 Gy (12.0–32.6 Gy)	97.4% at 15 m	25.2 m (6.5–51 m)
Pancreas cancer	Wieners (2015) [70]	Retrospective, single-centre	41 (55)	49	2.9 cm (1.0–7.3 cm)	18.1 Gy	91% at 12 m	8.6 m (1.5–55.3 m)
	Drewes (2019) [71]	Retrospective, single-centre	16 (62.5)	45	2.2 cm (1.0–11.2 cm)	21 Gy (5–29.1 Gy)	87% at 3.3 m median FU	8.9 m (3.1–29.3 m)
Esophageal carcinoma	Omari (2018) [78]	Retrospective, single-centre	11 (n/a)	21 met. (31× liver)	2.2 cm (0.7–6.8 cm)	20.1 Gy (10–25 Gy)	85.7% at median 6.3 m FU	13.7 m
Gastric or gastro-esophageal adenocarcinoma	Geisel (2012) [72]	Retrospective, single-centre	8 (50)	12	4.6 cm (1.4–6.8 cm)	21 Gy (5–29 Gy)	100% at 8.4 m (± 6.8 m FU)	n/a
	Omari (2019) [73]	Retrospective, single-centre	12 (83.3)	36	2 cm (1–10.2 cm)	19.9 Gy (5.4–22.5 Gy)	89% at 8.3 m median FU	11.4 m (4.3–47 m)
Melanoma	Bretschneider (2015) [79]	Retrospective, single-centre	14 (85.7)	52 met. (31 x liver)	1.5 cm (0.7–10.0 cm)	18.5 Gy (12–25.0 Gy)	90%	8.0 m
Mixed	Heinze (2020) [80]	Retrospective, single-centre	59 (71.1)	194	A: 1.6 cm (0.4–3.8 cm) B: 6 cm (4–13.9 cm)	17.1 Gy (5–29.1 Gy)	90.2% at 6 m median FU	n/a
	Kieszko (2018) [11]	Retrospective, single-centre	61 (55.7)	73	< 8 cm	13 Gy (7–20 Gy)	88.7% at 6 m 70.7% at 12 m	96.7% at 6 m 79.6% at 12 m
	Ricke (2004) [59]	Prospective, single-centre	20 (55)	19	Group A (Tu > 5 cm) mean 7.7 cm (5.5–10.8 cm) Group B (tumour≤5 cm) mean 3.6 cm (2.2–4.9 cm)	17 Gy (12–25 Gy)	87% at 6 m; 53% at 12 m	89% at 6 m; 83% at 12 m
	Ricke (2004) [35]	Prospective phase II, single-centre	36 (50)	37	7.7 cm (5.5–10.8 cm)		93% at 12 m	n/a
	Tselis (2013) [14]	Retrospective, single-centre	41 (75)	50	84 cm^3^ (38–1348 cm^3^)	20 Gy (7–32 Gy) bid	89% at 6 m 73% at 12 m	13.2 m (0.6–31.8 m)
	Walter (2021) [44]	Retrospective, single-centre	106 (49)	244	3.2 cm (0.8–13.5 cm)	19.3 Gy (7–27.9 Gy)	88.4% at 6 m 71.5% at 12 m	92.3% at 6 m 76.3% at 12 m
	Brinkhaus (2014) [81]	Prospective single-centre	69 (53.6)	96 (22 pat. nonHCC/CCC/CRC)	range 1–10 cm	range 10–20 Gy	n/a	62.3% at 12 m (better in noncirrhotic patients)
	Collettini (2013) [13]	Retrospective, single-centre	32 (44)	34 (50% nonCRC)	4.3 cm (1.3–10.7 cm)	20 Gy (prescription dose)	mean 17.3 m	20.24 m
	Sharma (2013) [82]	Prospective, single-centre	10 (50)	12	3.8 cm (2.7–7.0 cm)	19.6 Gy (9.3–83.2 Gy)	75% at 12 m	n/a
	Mohnike (2016) [83]	Retrospective, single-centre	192/42 (50xHCC, 16xCCC, 84CRC)	296	mCTV 36,7 ml, (13–78,8 ml)	16.4 Gy (5.9–31.2 Gy)	89% at 12 m	20.1 m

LC outcomes were heterogeneous. Some studies reported LC rates up to 97% at various time points—6, 12, 15, 18, and 36 months, e.g. [63], [64], [74], [77]. The most commonly reported time point was 12 months, with control rates ranging from 53% [35] to 94.6% [63]. High remission rates following interstitial HDR brachytherapy were most frequently observed in liver metastases from neuroendocrine carcinomas, breast cancer, anal carcinoma, and renal cell carcinoma. In contrast, studies including heterogeneous tumour cohorts or lesions with unfavourable prognostic features—such as large size or difficult location—demonstrated lower LC rates, as illustrated by the 53% LC reported in the 2004 study by Ricke et al. [59].

Melanoma liver metastases demonstrated a favourable initial tumour response, with a reported median local remission rate of 90% [79]. However, due to the typically widespread metastatic pattern in melanoma, this high LC did not translate into prolonged OS (median OS 8 months).

Multiple studies, including those by Heinze et al. [80] and Ricke et al. [59], have demonstrated a clear correlation between tumour size, anatomical location, and brachytherapy efficacy. Reported median OS rates in these cohorts ranged from 8 months [79] to 51.2 months, the latter observed in patients with neuroendocrine carcinoma.

In summary, interstitial HDR brachytherapy appears to be a highly effective local treatment option for secondary liver metastases of non-colorectal origin. Both LC and OS outcomes are influenced by tumour type, lesion size, and anatomical location. Metastases from neuroendocrine tumours, GIST, breast, renal, and anal carcinomas seem to particularly benefit from this approach.

## 5 Special considerations

### 5.1 Oligometastatic setting and repeated treatment

Interstitial HDR liver brachytherapy has garnered increasing recognition as a flexible and effective treatment modality across various metastatic disease settings, particularly in patients presenting with oligometastatic disease (OMD). OMD is typically defined as the presence of up to five metastatic lesions. According to the EORTC/ESTRO consensus classification, nine distinct clinical scenarios of OMD exist, grouped into three main categories: de novo, repeat, and induced OMD [84].

The repeat OMD setting refers to patients who, after an initial diagnosis of oligometastatic disease, subsequently experience limited progression, oligorecurrence, or oligopersistence. Clinical data on interstitial HDR liver brachytherapy in this specific context remain scarce. However, Bilski et al. recently reported a substantial real-world cohort comprising 127 patients with repeat oligoprogression from CRC treated with interstitial brachytherapy. The median OS and PFS were 16 and 9 months, respectively. The 1-year PFS rate was 28%, while 1- and 2-year OS rates were 65% and 16%, respectively. Notably, patients with liver-only metastases who achieved an objective response exhibited significantly longer PFS. The treatment demonstrated a favourable safety profile, with an acute rate of grade 3 adverse events of 4.0% and no late toxicities reported [30].

In the context of induced OMD, which arises following the conversion of polymetastatic disease into a limited metastatic state through systemic therapy, interstitial HDR brachytherapy plays a crucial role in managing oligoprogressive, oligorecurrent, or oligopersistent disease. These subtypes describe scenarios of limited metastatic progression during ongoing systemic therapy, recurrence following complete remission, or persistent but stable lesions with minimal responsiveness to ongoing treatment, respectively. In such cases, the therapeutic objective is to delay systemic progression and prolong PFS, particularly in patients requiring a treatment holiday due to cumulative toxicity or quality-of-life concerns. A recent study by Cisek et al. demonstrated the efficacy of interstitial HDR brachytherapy in 68 patients with induced oligopersistent liver metastases. Patients who achieved an objective response experienced significantly longer OS and PFS. The median OS and PFS were 16 and 13 months, with 1- and 2-year OS rates of 77% and 18% and PFS rates of 51% and 5%, respectively [31]. In a complementary cohort focusing on induced oligoprogression, the same group reported median OS and PFS of 17 and 10 months, respectively, with 1- and 2-year OS rates of 69% and 25% and PFS rates of 45% and 2%. Notably, treatment was well tolerated, with G3 side effects reported in only 1% of patients [32].

Beyond oligometastatic settings, interstitial HDR brachytherapy may serve a palliative role in polymetastatic disease, particularly when liver metastases exhibit dominant progression or become symptomatic. This approach provides effective LC while preserving the feasibility of subsequent systemic therapies. Its advantages include minimally invasive application, short treatment duration, and brief hospitalization, typically limited to 48 h. Patient selection in these cases should be based on a multidisciplinary assessment, considering general condition, therapeutic intent, and an estimated life expectancy of at least 4–6 months.

Finally, liver reirradiation represents an expanding clinical indication for interstitial HDR brachytherapy. Dosimetric studies have consistently shown that CT-guided interstitial HDR brachytherapy may offer improved target volume coverage, and better sparing of uninvolved liver tissue and adjacent OoI compared to stereotactic body radiotherapy (SBRT) [3], [85], [86], [87]. These attributes make interstitial HDR brachytherapy a particularly suitable option in radiorecurrent tumours, including type 1 and type 2 reirradiation scenarios as defined by ESTRO/EORTC guidelines, provided that cumulative OoI dose constraints are strictly respected [88].

## 6 Toxicities and Adverse event management

From a technical perspective, interstitial brachytherapy represents a combination of minimally invasive interventional techniques and radiation therapy. As a result, complications may arise either from the catheter-based puncture procedure or from the application of therapeutic radiation. The overall complication profile of brachytherapy has been extensively studied. All published data support the conclusion that liver brachytherapy is a safe treatment modality, with major and minor complication rates averaging below 4% and 6%, respectively. The largest investigation of complication rates and risk factors was conducted by Mohnike et al., who analysed 343 interventions in 192 patients. They reported a major complication rate of 4.1%, comparable to rates observed with thermal ablation. Specific complications included grade III–IV haemorrhage (1.46%), ascites (0.29%), gastroduodenal ulceration (0.87%), radiation-induced liver disease (RILD) (0.5%), liver abscess (1.17%), and bile duct obstruction (0.29%). Minor complications included grade I haemorrhage (3.21%), ascites (0.71%), pleural effusion (12.2%), and pneumothorax (1.75%). The most frequently reported patient symptoms were pain and nausea, which were typically transient and manageable [83].

Bleeding remains a clinically relevant risk, particularly during or immediately after catheter placement. Severe haemorrhagic events have been shown to correlate strongly with advanced liver cirrhosis—especially in patients with HCC—and with the use of low-molecular-weight heparin during the peri-procedural period. Thrombocytopenia is another significant factor, with low platelet counts associated with both minor and major bleeding events, independent of the number of implanted catheters [16], [83], [89]. While bleeding complications graded as CTCAE ≥3 are uncommon, they represent a serious risk and may be fatal in rare cases. Therefore, strict adherence to indication criteria in patients with cirrhosis, careful periprocedural management, and monitoring of coagulation parameters are essential —particularly in those with platelet counts below 90,000/μL. Whenever possible, anticoagulation therapy should be temporarily suspended around the time of the intervention. The prophylactic use of anticoagulants, such as low-molecular-weight heparin, is contraindicated prior to or during brachytherapy procedures [89].

One of the key clinical advantages of brachytherapy is its ability to spare healthy liver tissue. Unlike EBRT techniques, classical RILD has not been reported in the context of liver brachytherapy. Instead, isolated cases of atypical liver side effects—such as transient icteric enzyme elevation or mild ascites—have been observed [83]. Among extrahepatic organs at risk, the stomach and duodenum are most vulnerable to clinically significant adverse events, particularly gastroduodenal ulceration when exposed to D1 cm^3^ radiation doses exceeding 14–15 Gy. Therefore, high-dose proton-pump inhibitors should be prescribed for 6–8 weeks when dose exposure to these structures is expected to approach or exceed these thresholds [50], [83].

Tumour seeding along the puncture tract after CT-guided HDR brachytherapy is rare. In a large retrospective series of 1765 procedures, Büttner et al. observed puncture-tract seeding in 1.5% of interventions, with no significant difference between irradiated and non-irradiated puncture tracts [90]. In the rare event of recurrence along the tract, OS is not affected. In fact, local recurrences along the tract may be amenable to local salvage treatment with brachytherapy or SBRT [45], [91]. Some institutions nevertheless perform puncture-tract irradiation with approximately 5 Gy as a preventive measure. However, current evidence does not demonstrate a clear reduction in seeding risk.

## 7 Follow-up

### 7.1 General considerations

Patients undergoing interstitial HDR brachytherapy of the liver are most often in an oligometastatic stage of disease. This clinical state—characterized by limited metastatic spread—represents an intermediate phase between purely localised and widespread systemic disease and is generally regarded as amenable to local treatment. The concept applies universally across local therapeutic modalities, including resection, SBRT, thermal ablation, and brachytherapy. As a result, clinical decision-making regarding subsequent treatment is typically guided by the same principles as those applied during initial treatment planning.

The selection of local therapy should always be based on an individualised oncologic assessment, which incorporates factors such as patient age, comorbidities, previous therapies, and the anticipated therapeutic benefit versus treatment-related burden. In the follow-up of patients treated with interstitial brachytherapy, the primary aim is not only to monitor for disease progression but also to ensure timely identification of any potential for further effective local interventions. Once local options have been exhausted, prompt re-initiation of systemic therapy should remain a central element of comprehensive disease management.

### 7.2 Imaging and special considerations

Follow-up imaging should be performed at intervals of no more than three months and, in some cases, earlier depending on clinical context. The preferred imaging modality is MRI using a hepatocyte-specific contrast agent such as Gd-EOB-DTPA (Primovist, Bayer, Germany), which offers high sensitivity for the detection of new liver lesions and reliable assessment of post-treatment changes [51], [92], [93]. However, in patients with advanced liver cirrhosis, image quality may be compromised due to impaired contrast uptake in normal liver tissue.

Radiation effects in healthy liver parenchyma are often visible as areas of reduced contrast uptake on MRI with Gd-EOB-DTPA. These regions of decreased liver function can become apparent within a few days of treatment and typically reach their maximal extent at around six weeks post-intervention. The extent and location of these areas correlate directly with delivered radiation dose and can be precisely delineated in imaging, provided the dosimetric data are known [51], [92], [93].

It is essential for radiologists and clinicians interpreting follow-up images to be familiar with these characteristic changes to avoid mistaking post-treatment effects for progressive disease. Over time, most of these functionally impaired regions gradually resolve, with liver function typically recovering within six months. During early follow-up, it can be difficult to differentiate between post-therapeutic changes and new tumour foci within treated zones, particularly if the entire lesion did not receive a full ablative dose. In such cases, diffusion-weighted MRI sequences often provide valuable additional information [56], [94]. Response after interstitial brachytherapy can be delayed, and later imaging assessment may improve prognostic discrimination [94]. Histopathological data further suggest ongoing radiation-induced tumour cell death over time and support the use of the LI-RADS Radiation Treatment Response Algorithm for response assessment after HDR brachytherapy [57].

When MRI is contraindicated or not feasible, multiphase contrast-enhanced CT may be used as an alternative for liver imaging. This should be complemented by a clinical examination and laboratory testing, including tumour markers where clinically appropriate. In patients with extrahepatic primary tumours, further diagnostic work-up may be required based on the nature of the disease. Recommendations from existing disease-specific guidelines should also be consulted, especially regarding whole-body staging intervals.

In specific situations—such as when clinical suspicion for recurrence exists but findings on MRI or CT are inconclusive— PET/CT may be indicated. Depending on the tumour entity, different tracers can be used, such as FDG, DOTATOC, or FAPI, to help identify either intrahepatic recurrence or extrahepatic disease sites not detectable by standard imaging.

## 8 Conclusions

In summary, interstitial HDR brachytherapy is an effective, and highly conformal local treatment for selected primary and secondary liver tumours. Its precise dose delivery, minimal side effects, and suitability for otherwise ineligible patients make it an important component of multidisciplinary liver tumour management.

This ESTRO consensus recommendations provide comprehensive and standardised guidance on patient selection, image-guided catheter implantation, treatment planning, and follow-up. By harmonising technical approaches and reporting standards across institutions, these guidelines aim to improve treatment quality and safety while facilitating meaningful comparison of clinical outcomes. Standardisation through these recommendations will also support future prospective research and multicentre data collection. Such efforts are essential to further refine patient selection, optimise dose concepts, and strengthen the evidence base for interstitial HDR liver brachytherapy.

## 9 Disclaimer

ESTRO guidelines present scientific and medical opinions for educational and informational purposes only. Commercial use of any content in the guidelines without the prior written consent of ESTRO is strictly prohibited. The guidelines are not, and nor are they intended or implied to be, a substitute for professional medical advice or medical care. The advice of a medical professional should always be sought prior to commencing any form of medical treatment. Under no circumstances will ESTRO be held liable for any decision or action taken as a result of reliance on the content of the guidelines. ESTRO and all of its staff, agents and members disclaim any and all warranties and representations (implied or otherwise) with regards to the information contained in the guidelines. Each guideline is based on information available at the time the corresponding writing panel conducted its research and discussions on the topic. Guidelines should neither be deemed inclusive of all proper methods of care or factors influencing the decision making, nor exclusive of other methods reasonably directed to obtaining the same results. There may be new developments since publication that are not reflected in any one guideline and that may, over time, form the basis for ESTRO to revisit and update the guideline.

## Funding

This research did not receive any funding.

## Declaration of competing interest

The authors declare that they have no known competing financial interests or personal relationships that could have appeared to influence the work reported in this paper.
